# Task Difficulty Modulates the Impact of Emotional Stimuli on Neural Response in Cognitive-Control Regions

**DOI:** 10.3389/fpsyg.2012.00345

**Published:** 2012-09-12

**Authors:** Agnes J. Jasinska, Marie Yasuda, Rebecca E. Rhodes, Cheng Wang, Thad A. Polk

**Affiliations:** ^1^Michigan Institute for Clinical and Health Research, University of MichiganAnn Arbor, MI, USA; ^2^Department of Psychology, University of MichiganAnn Arbor, MI, USA

**Keywords:** emotion, cognitive control, executive function, emotion-cognition interactions, fMRI, ACC, DLPFC, IFG

## Abstract

Both heightened reactivity to emotional stimuli and impaired cognitive control are key aspects of depression, anxiety, and addiction. But the impact of emotion on cognitive-control processes, and the factors that modulate this impact, are still not well understood. We examined the effects of threat and reward distracters on the neural correlates of cognitive control using functional MRI (fMRI) and the Multi-Source Interference Task (MSIT). Behaviorally, subjects were slower and less accurate on the more demanding incongruent trials compared to the easier congruent trials. In addition, both threat and reward distracters significantly impaired the speed of responding on incongruent trials relative to the no-distracter condition. At the neural level, we used the *incongruent – congruent* contrast to functionally define four cognitive-control regions of interest (ROIs): anterior cingulate cortex (ACC), left and right inferior frontal gyrus (IFG)/insula, and right dorsolateral prefrontal cortex (DLPFC). A repeated-measures analysis of variance on the extracted contrast values in these ROIs indicated a significant interaction of stimulus salience and task difficulty on the neural response in cognitive-control regions. Specifically, threat distracters significantly *decreased* the response in cognitive-control regions on incongruent trials, whereas they significantly *increased* that response on congruent trials, relative to the no-distracter condition. Exploratory analyses of the amygdala response showed a similar interaction of stimulus salience and task difficulty: threat distracters significantly *decreased* the amygdala response only on incongruent trials. Overall, our results suggest that the impact of emotional distracters on the neural response in cognitive-control regions as well as in the amygdala is modulated by task difficulty, and add to our understanding of the factors that determine whether emotion enhances or impairs cognition.

## Introduction

Cognitive control is broadly defined as the ability to carry out a task despite interference from task-irrelevant stimuli, and it is a critical requirement for goal-directed behavior. Theoretical accounts have attributed cognitive-control functions to the prefrontal cortical regions (Miller and Cohen, 2001). More specifically, functional MRI (fMRI) evidence has shown that cognitive-control functions rely on a distributed cortical network, including the anterior cingulate cortex (ACC) extending into the dorsomedial prefrontal cortex (DMPFC) along the medial wall, and the dorsolateral prefrontal cortex (DLPFC) and inferior frontal gyrus (IFG) extending into the insula laterally, as well as parietal regions (for review, see Duncan and Owen, 2000; for meta-analyses, see Nee et al., 2007; Niendam et al., 2012). However, this evidence reflects cognitive-control processes recruited primarily in the absence of emotionally salient stimuli (e.g., the classical Stroop task with emotionally neutral color words), and thus leaves open the question of whether – and how – these cognitive-control processes are modulated by emotional salience.

One approach to investigating the relationship between cognitive control and emotion has been to modify an existing cognitive-control task to include emotionally salient stimuli, for example, as task-irrelevant distracters or task-relevant targets. These emotionally salient stimuli can be either negative (threat-related) or positive (reward-related) in valence; they can vary in modality (e.g., visual or auditory) and form (e.g., images vs. words); and they can be presented simultaneously with, or precede, task targets. Several neuroimaging studies have examined the effects of threat-related negative emotional stimuli on the neural correlates of cognitive control in a variety of interference tasks (Whalen et al., 1998a; Compton et al., 2003; Bishop et al., 2004; Etkin et al., 2006; Blair et al., 2007; Egner et al., 2008; Kanske and Kotz, 2011a,b; Hu et al., 2012), working-memory tasks (Dolcos and McCarthy, 2006; Dolcos et al., 2006, 2008; Anticevic et al., 2010; Shafer and Dolcos, 2012), and categorization tasks (Gu et al., 2012; Shafer et al., 2012). Taken together, these studies support the notion that negative emotional stimuli modulate activity in the cognitive-control network, as well as in the amygdala and ventral ACC, although the magnitude and direction of this modulation differs across studies, tasks, and individuals. Although less studied, modulatory effects of reward-related positive emotional stimuli on the cognitive-control network have also been reported, with similarly inconclusive results (Blair et al., 2007; Padmala and Pessoa, 2010; Savine and Braver, 2010; Krebs et al., 2011). Finally, at the behavioral level, both positive and negative emotional stimuli have been shown to sometimes enhance (Kanske and Kotz, 2011a,b,c) and sometimes impair (Blair et al., 2007; Gu et al., 2012; Jasinska et al., 2012b) cognitive control. Furthermore, significant behavioral effects of emotional stimuli have been sometimes observed only in the more demanding task conditions (Kanske and Kotz, 2011a,b; Gu et al., 2012) and other times only in the less demanding task conditions (Hu et al., 2012; Shafer et al., 2012). Thus, overall, the existing evidence suggests that the impact of emotionally salient stimuli on cognitive-control processes and on cognitive task performance is modulated by other factors (Cohen and Henik, 2012). But most of these factors and their neural mechanisms of action are still poorly understood.

In our previous behavioral investigation (Jasinska et al., 2012b), we examined *task difficulty* (also referred to as *task load* or *task demands*) as a plausible factor modulating the impact of threat distracters on cognitive task performance (Gu et al., 2012; Hu et al., 2012; Shafer et al., 2012). We used the Multi-Source Interference Task (MSIT; Bush and Shin, 2006), a demanding cognitive interference task with robust neural and behavioral effects, in order to maximize the chances that modulation of these task effects by threat stimuli could be detected. The task included threat distracters (angry and fearful faces) as well as perceptually matched neutral distracters (neutral faces), in addition to the no-distracter condition, and threat distracters were rated as significantly higher in both emotional intensity and distractability than neutral distracters by the subjects. Our behavioral data indicated a significant interaction between stimulus salience and task difficulty (i.e., the easier congruent MSIT condition vs. the more demanding incongruent MSIT condition) in both measures of task performance. In particular, relative to both the neutral-distracter and no-distracter conditions, threat distracters *impaired* task performance on the more demanding incongruent trials, on which a correct response required overcoming interference from a competing response tendency; but threat distracters actually *enhanced* task performance on the easier congruent trials, which relied on a simple stimulus-response mapping.

Having previously demonstrated robust behavioral effects of threat distracters on cognitive task performance, the goal of the current study was to investigate the neural processes that underlie these effects. We employed event-related fMRI, which measures the blood-oxygenation-level-dependant (BOLD) signal considered to be an index of neural activity, and a novel version of the MSIT modified to include both threat and reward distracters. The primary aim of our study was to examine the impact of threat and reward distracters on the neural response of cognitive-control regions (including the ACC, DLPFC, and IFG/insula) during cognitive task performance. Based on the results of our behavioral study (Jasinska et al., 2012b), we expected to observe an interaction of stimulus salience and task difficulty, such that threat distracters should decrease the response in cognitive-control regions in the more demanding incongruent MSIT condition, but increase the response in cognitive-control regions in the easier congruent MSIT condition. We also tentatively hypothesized that a similar interaction of stimulus salience and task difficulty on the response of cognitive-control regions would be observed for reward distracters. The secondary, more exploratory aim of our study was to test whether a similar interaction of stimulus salience and task difficulty would be observed in the amygdala for threat and reward distracters.

## Materials and Methods

### Subjects

Fifteen healthy Caucasian females aged 20 to 31 years (M = 24.4 years, SD = 3.4 years) participated in the study. Due to technical problems and loss of data for two participants, we present the fMRI data from the final sample of 13 participants. All subjects were right-handed and had normal or corrected-to-normal vision. Exclusion criteria included any serious medical condition, head injury or trauma, lifetime diagnosis of psychiatric illness, current use of a psychoactive medication, and cigarette smoking. All subjects had participated in a behavioral investigation using the threat-distracter MSIT approximately 2 years prior to the fMRI experiment (Jasinska et al., 2012b). Only females were included at this stage to maximize statistical power to detect the effects of interest in light of documented sex differences in the processing of emotional stimuli in the brain (Klein et al., 2003; Wrase et al., 2003). The study was approved by the University of Michigan Medical School IRB and all subjects provided written informed consent.

### Threat- and reward-distracter MSIT

**Figure 1:**
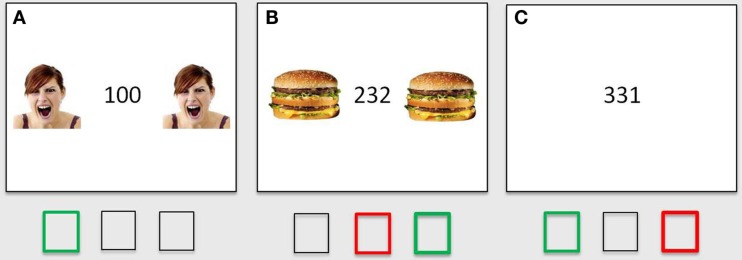
**Threat- and reward-distracter MSIT**. MSIT congruent trial with flanker threat distracters **(A)**, MSIT incongruent trial with flanker reward distracters **(B)**, and MSIT incongruent trial with no distracters (so only emotionally neutral stimuli; **C**). The correct responses are shown in green; common incorrect responses on incongruent trials (i.e., indicating the position instead of the value of the oddball number) are shown in red.

We employed a modified version of the MSIT (Bush et al., 2003; Bush and Shin, 2006). The MSIT combines the sources of interference from Erikson, Stroop, and Simon tasks, and it was designed to elicit activation in the prefrontal cortical regions associated with interference processing, particularly the dorsal ACC, in neuroimaging studies (Bush et al., 2003). On each trial, subjects were presented with a row of three numbers ranging from 0 to 3, and one of the numbers was different from the other two (the oddball number). Subjects were instructed to indicate the identity of the oddball number with a corresponding key press on a scanner-compatible response glove: a key press with the index finger if the oddball number was “1,” with the middle finger if the oddball number was “2,” and with the ring finger if the oddball number was “3.” On congruent trials, the identity of the oddball number corresponded to its location and the other two numbers were 0’s, not related to any valid key press response (e.g., “1” on the left and two zeros, or two zeros and “3” on the right). On incongruent trials, the identity of the oddball number was incongruent with its position and the other two numbers were associated with competing key press responses (e.g., “3” on the left” or “1” on the right), resulting in stimulus-response incompatibility and response interference. The incongruent condition vs. congruent condition contrast was used as a measure of interference in reaction times (*incongruent RT – congruent RT*) and in accuracy (*congruent accuracy – incongruent accuracy*). We modified the MSIT to include threat and reward flanker distracters in addition to the no-distracter condition (Figure 1). Threat distracters were color images of human faces signaling the presence of a threat (angry or fearful expression). The majority of face stimuli (13 images) were selected from standardized sets (Gur et al., 2002; Tottenham et al., 2009), supplemented with a small number of carefully selected stimuli from online sources (three images). Angry and fearful faces displayed intense emotion and showed bared teeth and/or open mouth as an additional perceptual homogeneity criterion. Reward distracters were color images of high-calorie, highly palatable foods selected from online sources. On distracter trials, two identical distracter images flanked the MSIT stimuli on both sides. Neutral distracters were not included in the fMRI paradigm in order to minimize the number of trials and the duration of the scanning protocol in light of documented habituation of the amygdala response to repeated presentation of threat stimuli (Breiter et al., 1996; Whalen et al., 1998b; Wright et al., 2001), which could potentially reduce or even eliminate threat-distracter effects over time; and because our previous behavioral study already established a significant effect of threat distracters (angry or fearful faces) above and beyond that of closely matched neutral distracters (neutral faces; see also the Discussion). Following instructions and a short practice, subjects completed two runs of the MSIT, 60 trials per run, for a total of 120 trials (20 congruent/threat distracters, 20 incongruent/threat distracters, 20 congruent/reward distracters, 20 incongruent/reward distracters, 20 congruent/no distracters, 20 incongruent/no distracters). On each trial, task stimuli together with distracters were presented for 1 s, followed by a white screen for another 1 s, a fixation cross of jittered duration (mean 6 s, range 4–10 s), and another white screen for 300 ms. The total response limit on each trial was 2 s. Including the jitter, the task took approximately 17 min to perform.

### fMRI data acquisition and preprocessing

Scanning was performed on a 3T GE Signa Excite 2 scanner (Milwaukee, Wisconsin), beginning with a structural T1-overlay image [repetition time (TR) = 250 ms, echo time (TE) = 5.7 ms, flip angle (FA) = 85°, field of view (FOV) = 220 mm, 43 oblique axial slices, 256 × 256, slice thickness 3.0 mm]. Functional scans were collected using a T2*-weighted spiral-in acquisition sequence (gradient echo, TR = 2000 ms, TE = 30 ms, FA = 90°, FOV = 220 mm, 64 × 64, slice thickness 3.0 mm; Noll et al., 1998). High-resolution T1 scans were also obtained for precise anatomical localization [3D spoiled-gradient echo (3D-SPGR) with inversion recovery prep, time of inversion = 400 ms, TR = 9.0 ms, TE = 1.8 ms, FA = 15 degrees, FOV = 260 mm, 128 slices, 256 × 256, 1.2 mm slice]. The functional scans were physio-corrected, slice-time-corrected, and realigned to the first scan using the MCFLIRT program (FSL Analysis Group, FMRIB, Oxford, UK). Subsequent processing was done using SPM 8 (Wellcome Institute of Cognitive Neurology, London, UK). For each subject, the high-resolution 3D-SPGR image was co-registered with a mean functional scan and anatomically normalized to the Montreal Neurological Institute (MNI) 152 template. The resulting transformation parameters were then applied to the co-registered functional volumes. All functional volumes were smoothed with a Gaussian kernel (8 mm^3^).

### fMRI data analyses

After preprocessing, the individual fMRI data were analyzed using a jittered event-related design in the framework of the General Linear Model as implemented in SPM8. Regressors of interest (i.e., vectors of the onset times specific to each trial type) were convolved with a canonical hemodynamic response function (HRF) with a time derivative to account for between-subject and between-voxel variability in the response peak. Six regressors of interest were defined for the MSIT task: MSIT incongruent/threat-distracter trials, MSIT incongruent/reward-distracter trials, MSIT incongruent/no-distracter trials, MSIT congruent/threat-distracter trials, MSIT congruent/reward-distracter trials, and MSIT congruent/no-distracter trials. A number of contrasts were estimated for each individual subject. First, the *incongruent/no distracters – congruent/no-distracters* contrast was used to identify brain regions associated with cognitive control. Significant clusters in the established cognitive-control regions (i.e., ACC, DLPFC, and IFG/insula) were then saved as functionally defined region of interest (ROI) masks. Next, several other contrasts of interests were estimated, including the *incongruent/threat distracters – fixation, incongruent/reward distracters – fixation*, and *incongruent/no distracters – fixation* contrasts. The incongruent condition was compared to a fixation baseline rather than to the congruent condition for two reasons: first, to allow a comparison of incongruent and congruent conditions against a common baseline; and second, to avoid “double-dipping” (i.e., testing the same contrast that was used to define the ROI). Group analyses were then conducted using random-effects models and one-sample *t*-tests in SPM8. Mean contrast values were extracted from each ROI mask for all contrasts of interest for all participants. These values were then analyzed using repeated-measures ANOVAs and *post hoc* tests in SPSS 19.0, starting with an omnibus ANOVA, in order to test for main and interactive effects of stimulus salience and task difficulty on the BOLD response in cognitive-control regions. Exploratory analyses were also conducted for the amygdala, using anatomically defined left and right amygdala masks. All *t*-tests were two-tailed paired-sample *t*-tests.

## Results

### Behavioral results

The behavioral results from the MSIT are summarized in Table 1. We first conducted a 2 × 3 repeated-measures ANOVA with two factors (factor 1: task difficulty: easier/congruent trials or more demanding/incongruent trials; factor 2: stimulus salience: threat distracters, reward distracters or no distracters/neutral stimuli) on correct RTs and accuracy rates. Consistent with previous reports (Bush et al., 2003; Bush and Shin, 2006), we found a significant MSIT interference effect (i.e., main effect of task difficulty or congruency) in both measures of task performance: in RTs, *F*(1, 14) = 224.184, *p* < 0.0001, and in accuracy, *F*(1, 14) = 22.232, *p* < 0.0001 (see Table 1). Namely, subjects were significantly slower to respond in the incongruent compared to the congruent condition, *t*(14) = 14.966, *p* < 0.0001, and they were also significantly less accurate in the incongruent compared to the congruent condition, *t*(14) = −4.709, *p* < 0.0001. We also found a significant main effect of stimulus salience in RTs, *F*(1, 14) = 12.385, *p* < 0.0001, but not in accuracy, *F*(1, 14) = 0.237, *p* = 0.790. Collapsing across the MSIT congruent and incongruent trials, subjects were significantly slower to respond in the presence of threat distracters, *t*(14) = 3.833, *p* = 0.002, or reward distracters, *t*(14) = 3.436, *p* = 0.004, compared to the no-distracter condition with only neutral stimuli. Speed of responding in the presence of threat and reward distracters did not differ, *t*(14) = 0.812, *p* = 0.43. Lastly, there was also a significant interaction of task difficulty and stimulus salience in RTs, *F*(2, 13) = 7.209, *p* = 0.003, but not in accuracy, *F*(2, 13) = 0.473, *p* = 0.628. Specifically, the distracter effects were very robust on the more demanding incongruent trials [threat-distracter RT > no-distracter RT, *t*(14) = 5.727, *p* < 0.0001; reward-distracter RT > no-distracter RT, *t*(14) = 5.021, *p* < 0.0001], but were virtually absent on the easier congruent trials (*p*s > 0.440), except for a trend toward significantly higher RTs in the congruent trials with threat distracters compared to the congruent trials with reward distracters, *t*(14) = 1.864, *p* = 0.083. The speed of responding on incongruent trials with threat distracters compared to reward distracters did not significantly differ, *t*(14) = −0.456, *p* = 0.655.

**Table 1:** **Summary of behavioral task results**.

Distracter	MSIT condition		*MSIT interference effect*		
	Congruent	Incongruent	*Mean*	*T*	*P value*
**RT (ms)**					
Threat	647 (109)	857 (105)	210 (72)	−11.266	<0.0001
Reward	632 (98)	861 (118)	229 (63)	−13.989	<0.0001
Null	637 (101)	807 (107)	170 (53)	−12.544	<0.0001
Overall	639 (100)	842 (109)	203 (53)	−14.966	<0.0001
**ACCURACY (PROPORTION ACCURATE)**					
Threat	0.990 (0.039)	0.913 (0.090)	0.077 (0.062)	4.766	<0.0001
Reward	0.983 (0.052)	0.933 (0.084)	0.050 (0.078)	2.485	0.026
Null	0.990 (0.028)	0.927 (0.116)	0.063 (0.097)	2.523	0.024
Overall	0.988 (0.081)	0.925 (0.081)	0.063 (0.052)	4.709	<0.0001

### Neuroimaging results

#### Identifying cognitive-control regions with the incongruent – congruent contrast

**Figure 2:**
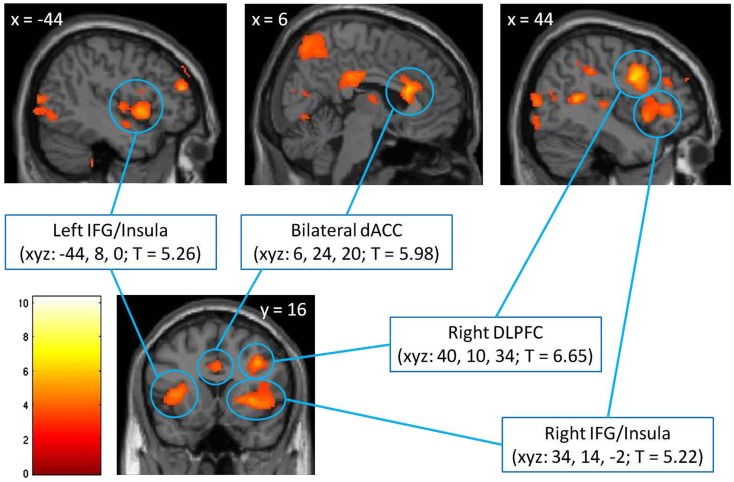
**Identification of cognitive-control regions in the brain, as assessed with a comparison of voxel-wise responses to incongruent and congruent MSIT trials in the absence of emotional distracters (the *incongruent/no distracters – congruent/no distracters* contrast), thresholded at *p* < 0.001, minimum 10 contiguous voxels**. The significant clusters are localized using Montreal Neurological Institute (MNI) coordinates of left/right (*x*), anterior/posterior (*y*), and superior/inferior (*z*), respectively, and are shown against the MNI anatomical brain template. The scale represents *t* values. dACC, dorsal anterior cingulate cortex; DLPFC, dorsolateral prefrontal cortex; IFG, inferior frontal gyrus.

In the first step, we identified brain regions associated with cognitive control by directly comparing the response to incongruent and congruent trials in the absence of emotionally salient stimuli (the *incongruent/no distracters – congruent/no distracters* contrast, thresholded at *p* < 0.001, minimum 10 contiguous voxels). This comparison yielded robust activation in regions previously associated with cognitive control, including bilateral anterior cingulate (ACC), left and right IFG/insula, and right DLPFC (Figure 2), as well as a number of other cortical and subcortical regions (Table 2). Significant clusters in the ACC, left IFG/insula, right IFG/insula, and right DLPFC were saved as functionally defined cognitive-control ROIs. In subsequent analyses, we used contrast values extracted from these four ROIs in order to test for main and interactive effects of stimulus salience and task difficulty on neural correlates of cognitive control.

**Table 2:** **Response to incongruent trials relative to congruent trials in the absence of emotionally salient distracters**.

Region	BA	*x*	*y*	*z*^a^	*k*^b^	*T*	*Z*^c^
R Precuneus	7	30	−64	26	877	10.32	5.15
**R Middle Frontal Gyrus (DLPFC)**	9, 6	40	10	34	538	6.65	4.23
L Middle Frontal Gyrus	10	−48	46	20	47	6.49	4.18
**Anterior Cingulate (ACC)**	24	6	24	20	175	5.98	4.00
L Precentral Gyrus	6	−30	−10	30	145	5.87	3.96
R Superior Temporal Gyrus		46	−50	12	53	5.66	3.88
Middle/Posterior Cingulate	23, 31	−2	−28	32	294	5.61	3.86
**L IFG/Insula**	13, 47	−44	8	0	159	5.26	3.72
**R IFG/Insula**	47, 13	34	14	−2	245	5.22	3.70
R Precuneus	7	12	−74	34	28	5.00	3.61
**R Superior Frontal Gyrus**	9	34	52	36	38	4.89	3.56
L Superior Occipital Gyrus	31	−28	−62	20	30	4.57	3.41
L Thalamus (Pulvinar)		−18	−22	18	31	4.51	3.38
L Middle Occipital Gyrus	19	−26	−88	18	25	4.47	3.37
R Calcarine Sulcus	18	14	−82	16	14	4.44	3.35
R Thalamus (Pulvinar)		18	−28	12	15	4.41	3.33
R Thalamus		14	−10	16	26	4.40	3.33
R Precuneus		24	−58	52	12	4.36	3.31

*This contrast was used to functionally identify cognitive-control regions (specifically, the ACC, DLPFC, and IFG/insula, shown in bold) for subsequent analyses*.

*BA, Brodmann Area; DLPFC, dorsolateral prefrontal cortex; L, left; R, right*.

*^a^Stereotactic coordinates of the peak voxel from the Montreal Neurological Institute atlas, left/right (*x*), anterior/posterior (*y*), and superior/inferior (*z*), respectively*.

*^b^Spatial extent of the cluster in voxels (minimum 10 contiguous voxels)*.

*^c^Significance threshold of *p* < 0.001*.

#### Interaction of stimulus salience and task difficulty on neural correlates of cognitive control

**Figure 3:**
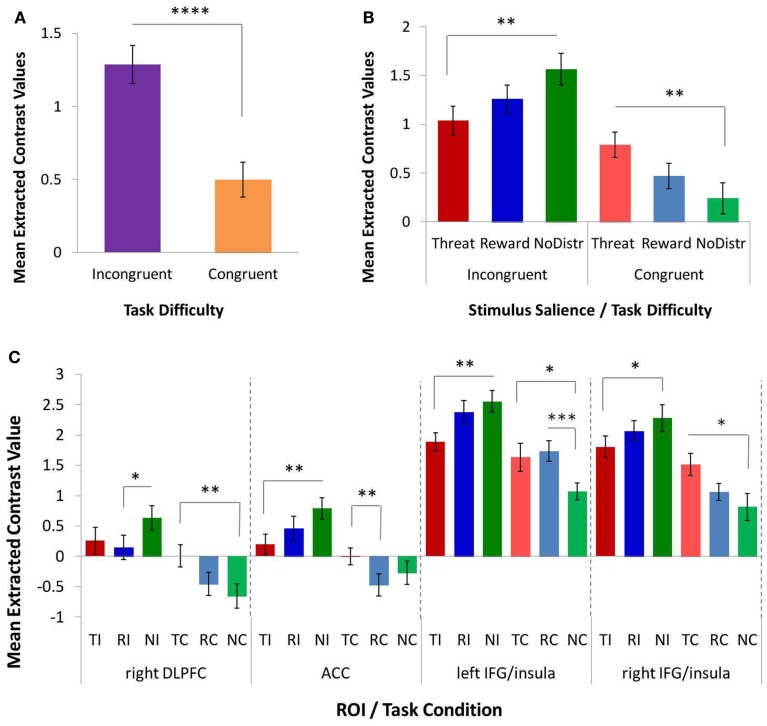
**The effects of task difficulty and stimulus salience on the response in functionally defined cognitive-control regions, as assessed with a repeated-measures ANOVA and *post hoct*-tests on extracted contrast values**. **(A)** A significant main effect of task difficulty (or MSIT condition) on the response in cognitive-control regions. The more demanding incongruent condition elicited a greater response than the easier congruent condition. **(B)** A significant interaction of task difficulty and stimulus salience on the response in cognitive-control regions. Threat distracters reduced the response to incongruent trials, but increased the response to congruent trials, relative to the no-distracter condition. **(C)** Interaction of task difficulty and stimulus salience in individual cognitive-control ROIs: right DLPFC, ACC, left IFG/insula, and right IFG/insula. ACC, anterior cingulate cortex; DLPFC, dorsolateral prefrontal cortex; IFG, inferior frontal gyrus; MSIT, Multi-Source Interference Task; ROI, region of interest; NC, congruent/no distracters; NI, incongruent/no distracters; RC, congruent/reward distracters; RI, incongruent/reward distracters; TC, congruent/threat distracters; TI, incongruent/threat distracters. *p < 0.10; **p < 0.05; ***p < 0.01; ****p < 0.001.

The omnibus 4 × 2 × 3 repeated-measures ANOVA on extracted contrast values (factor 1, ROI: ACC, left IFG/insula, right IFG/insula, right DLPFC; factor 2, task difficulty: congruent and incongruent; and factor 3, stimulus salience: threat distracters, reward distracters, and no distracters/neutral stimuli) yielded several significant main and interactive effects. The results are shown in Figure 3. Consistent with robust behavioral MSIT effects, we observed a significant main effect of task difficulty (i.e., main effect of congruency) on the BOLD response in cognitive-control regions, *F*(1, 12) = 30.480, *p* < 0.0001, with the response to incongruent trials significantly higher than that to congruent trials (Figure 3A). There was also a significant main effect of ROI, *F*(3, 10) = 11.870, *p* = 0.001, as well as a significant interaction of ROI and stimulus salience, *F*(6, 7) = 11.392, *p* = 0.003, on the BOLD response in cognitive-control regions. Critically, and consistent with our main hypothesis, we found a significant interaction of task difficulty and stimulus salience on the BOLD response in cognitive-control regions, *F*(2, 11) = 4.498, *p* = 0.037 (Figure 3B). Specifically, we found a significant double dissociation with respect to threat-distracter effects on the BOLD response in cognitive-control regions across the two levels of task difficulty: threat distracters significantly *decreased* the BOLD response in cognitive-control regions on the more demanding, incongruent MSIT trials, *t*(12) = −2.343, *p* = 0.037, whereas they significantly *increased* the BOLD response in cognitive-control regions on the easier, congruent MSIT trials, *t*(12) = 2.247, *p* = 0.044, relative to the no-distracter condition. Reward distracters produced levels of response intermediate between threat-distracter and no-distracter conditions, but these effects did not reach significance (*p*s > 0.170). The difference in the BOLD response in cognitive-control regions between threat and reward distracters was also not significant (*p*s > 0.173).

To further investigate the observed effects, separate 2 × 3 repeated-measures ANOVAs were conducted for each ROI (Figure 3C). The ROI-specific ANOVAs confirmed a significant main effect of task difficulty on the BOLD responses in all four cognitive-control ROIs, with a higher response magnitude for incongruent compared to congruent trials in ACC, *F*(1, 12) = 16.372, *p* = 0.002; in right DLPFC, *F*(1, 12) = 29.664, *p* < 0.0001; in left IFG/insula, *F*(1, 12) = 15.540, *p* = 0.002; and in right IFG/insula, *F*(1, 12) = 40.427, *p* < 0.0001. Importantly, the ROI-specific ANOVAs also confirmed a significant interaction of task difficulty and stimulus salience on the BOLD response in ACC, *F*(2, 11) = 7.936, *p* = 0.007, and in left IFG/insula, *F*(2, 11) = 4.696, *p* = 0.034, as well as a trend toward a significant interaction in right DLPFC, *F*(2, 11) = 3.786, *p* = 0.056, and in right IFG/insula, *F*(2, 11) = 2.837, *p* = 0.101. Specifically, threat distracters produced significant decreases in the BOLD response to incongruent trials in ACC, *t*(12) = −2.861, *p* = 0.014, and in left IFG/insula, *t*(12) = −2.612, *p* = 0.023, as well as a trend toward a significant decrease in right IFG/insula, *t*(12) = −1.803, *p* = 0.097, relative to the no-distracter condition. Conversely, threat distracters produced a significant increase in the BOLD response to congruent trials in right DLPFC, *t*(12) = 2.699, *p* = 0.019, as well as a trend toward a significant increase in left IFG/insula, *t*(12) = 1.793, *p* = 0.098, and in right IFG/insula, *t*(12) = 1.915, *p* = 0.088, relative to the no-distracter condition. In addition, reward distracters produced a trend toward a significant decrease in DLPFC response to incongruent trials, *t*(12) = −1.888, *p* = 0.083, as well as a significant increase in left IFG/insula response to congruent trials, *t*(12) = 3.494, *p* = 0.004, relative to no distracters. No other effects of reward-distracters reached statistical significance. Threat distracters and reward distracters generally did not significantly differ in their effects on the BOLD response in cognitive-control regions, except for a trend toward a significant threat-related increase (i.e., a smaller decrease) in the ACC response to congruent trials relative to reward distracters, *t*(12) = 2.155, *p* = 0.052.

In summary, and consistent with our main hypothesis, we found a significant interaction of task difficulty and stimulus salience on the BOLD response in functionally defined cognitive-control regions. This interaction was driven by threat distracters, which had significant and dissociable effects on the response in cognitive-control regions depending on the level of task difficulty. Specifically, in the more demanding, incongruent MSIT condition, threat distracters acted to decrease the response in cognitive-control regions; in contrast, in the easier, congruent MSIT condition, threat distracters acted to increase the response in cognitive-control regions.

#### Exploratory analyses of amygdala response

**Figure 4:**
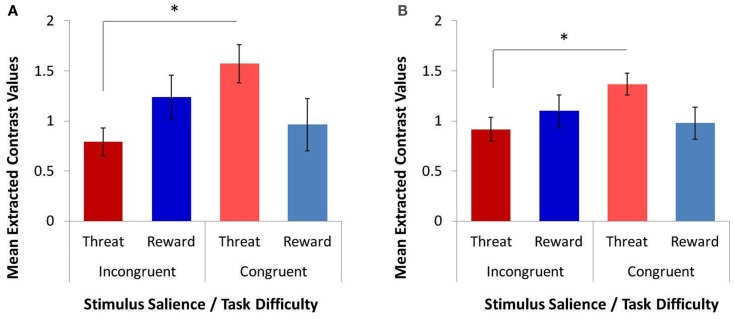
**The effects of task difficulty and stimulus salience on the response in anatomically defined left and right amygdala, as assessed with a repeated-measures ANOVA and post hoc *t-tests* on extracted contrast values**. We observed a significant interaction of task difficulty and stimulus salience on the response in both left amygdala **(A)** and right amygdala **(B)**. The amygdala response to threat distracters on the more demanding incongruent trials was significantly lower than to threat distracters on the easier congruent trials. In contrast, the amygdala response to reward distracters was not modulated by task difficulty. *p < 0.05.

In light of the documented importance of the amygdala in emotion processes and in emotion-cognition interactions, we also conducted exploratory analyses to test for main and interactive effects of stimulus salience and task difficulty on amygdala response. We used a 2 × 2 × 2 repeated-measures ANOVA (factor 1: ROI: left or right amygdala; factor 2: task difficulty: congruent or incongruent; factor 2: stimulus salience: threat distracters or reward distracters). The results are shown in Figure 4. There were no significant main effects of ROI and no interactions with ROI (*p*s > 0.251). As expected, the main effect of task difficulty on amygdala response was not significant, *F*(1, 12) = 1.091, *p* = 0.317. The main effect of stimulus salience was also not significant, *F*(1, 12) = 0.210, *p* = 0.655. However, we observed a significant interaction of task difficulty and stimulus salience on amygdala response, *F*(1, 12) = 4.992, *p* = 0.045. Specifically, averaging across left and right amygdala, the amygdala response to threat distracters was significantly reduced in the incongruent MSIT condition compared to the congruent MSIT condition, *t*(12) = −2.944, *p* = 0.012, whereas the amygdala response to reward distracters was not significantly affected by task difficulty, *t*(12) = 0.662, *p* = 0.520. The difference in the average amygdala response between threat distracters and reward distracters was not significant in either congruent or incongruent MSIT condition (*p*s > 0.134). This pattern of results was also observed in the left and right amygdala separately, as signaled by a lack of main or interactive effects of ROI. The left amygdala response to threat distracters on incongruent trials was significantly lower than to threat distracters on congruent trials, *t*(12) = −2.683, *p* = 0.020; and similarly, the right amygdala response to threat distracters on incongruent trials was significantly lower than to threat distracters on congruent trials, *t*(12) = −2.553, *p* = 0.025. In contrast, the responses to reward distracters in left and right amygdala were not significantly modulated by task difficulty (*p*s > 0.517). The responses to threat and reward distracters on either congruent or incongruent trials also did not significantly differ either in the left or right amygdala (*p*s > 0.112). In addition, correlations showed that the amygdala responses to threat and reward distracters were significantly positively correlated in all task conditions (left amygdala: incongruent trials, *r* = 0.831, *p* < 0.0001; congruent trials, *r* = 0.612, *p* = 0.026; right amygdala: incongruent trials, *r* = 0.774, *p* = 0.002; congruent trials, *r* = 0.602, *p* = 0.030).

To further explain this reduction in the amygdala response to threat distracters (but not to reward distracters) during task performance, we tested for correlations between the amygdala response and the response in the cognitive-control regions in congruent and incongruent MSIT conditions separately. In the threat-distracter condition, the amygdala response was not significantly correlated with the response in any of the four cognitive-control ROIs in either congruent or incongruent MSIT condition (*p*s > 0.148). However, on incongruent trials with reward distracters, the amygdala response was significantly positively correlated with the right IFG/insula response, *r* = 0.553, *p* = 0.050, and showed a trend toward a positive association with the left IFG/insula response, *r* = 0.516, *p* = 0.071. Interestingly, both reward-related associations were driven by the right amygdala (with left IFG/insula, *r* = 0.600, *p* = 0.030; with right IFG/insula, *r* = 0.558, *p* = 0.047); whereas the left amygdala response showed only a trend toward a positive association with the right IFG/insula response, *r* = 0.533, *p* = 0.061, and was not significantly associated with the left IFG/insula response, *r* = 0.444, *p* = 0.129. In addition, on congruent trials with reward distracters, the amygdala response was significantly negatively correlated with the right DLPFC response, *r* = −0.636, *p* = 0.019. The association with the right DLPFC was driven by the right amygdala, *r* = −0.735, *p* = 0.004, but was also detected at a trend level in the left amygdala, *r* = −0.550, *p* = 0.052.

## Discussion

Complex, bidirectional emotion-cognition interactions, including those between emotionally salient stimuli and cognitive-control processes, are crucial to goal-directed behavior and may be impaired in several psychological disorders such as depression, anxiety, and addiction. Although increasingly a research focus in the neurosciences, the neural mechanisms underlying the relationships between emotion and cognitive control are still incompletely understood. In particular, evidence suggests that emotionally salient stimuli can sometimes enhance and sometimes impair cognitive task performance, although our understanding of factors that determine which of the two effects occurs is still limited.

In the current fMRI study, building on the evidence from our previous behavioral investigation (Jasinska et al., 2012b) and from other studies (Gu et al., 2012; Hu et al., 2012; Shafer et al., 2012), we focused on *task difficulty* as a plausible factor modulating the impact of emotionally salient distracters on the response in cognitive-control regions and on cognitive task performance. We used an event-related fMRI design and a robust interference task, the MSIT (Bush and Shin, 2006), modified to include both threat and reward distracters. This permitted us to examine the main and interactive effects of stimulus salience (threat, reward, or neutral stimuli) and task difficulty (a demanding incongruent task condition vs. an easier congruent task condition).

As expected, the threat/reward-distracter MSIT produced robust behavioral effects. Consistent with prior studies using the standard MSIT (Bush et al., 2003), we found significant main effects of task difficulty (or congruency) in both RTs and accuracy: subjects were significantly slower and significantly less accurate in the more demanding incongruent condition compared to the easier congruent condition. Furthermore, these interference effects (incongruent vs. congruent contrasts) were present in all distracter conditions, supporting the notion that an additional cognitive-control process was required to overcome the interference on incongruent trials, which was not engaged (or engaged to a lesser degree) on congruent trials. We also found a significant main effect of stimulus salience, as well as a significant interaction of task difficulty and stimulus salience, in RTs but not in accuracy. Subjects were significantly slower in the presence of threat or reward distracters compared to no distracters, and this effect was driven by threat- and reward-distracter-related slowing specific to the incongruent trials but absent from the congruent trials. In contrast to our previous behavioral study (Jasinska et al., 2012b), we failed to observe an enhancing effect of threat distracters on task performance in the congruent condition in the behavioral data collected during fMRI. We also found no significant differences in RTs or accuracy between the threat- and reward-distracter conditions, suggesting comparable effects of both positive and negative emotional distracters on behavioral performance in our paradigm.

At the neural level, also as expected and consistent with previous fMRI studies using the standard MSIT (Bush et al., 2003), the *incongruent – congruent* contrast with no emotional stimuli yielded robust activation in regions associated with cognitive control: the bilateral ACC, left and right IFG/insula, and right DLPFC. We used this contrast to functionally define four cognitive-control ROIs for subsequent analyses, which were performed on extracted contrast values, with all six conditions of interest (incongruent/threat distracters, congruent/threat distracters, incongruent/reward distracters, congruent/reward distracters, incongruent/no distracters, and congruent/no distracters) compared to a common fixation baseline. But the key question addressed by our study was the impact of threat and reward distracters on the response in the cognitive-control ROIs, and whether this impact was modulated by task difficulty. Indeed, consistent with our main hypothesis, we found a significant interaction of stimulus salience and task difficulty on the response in cognitive-control regions. Specifically, and in agreement with our previous behavioral report (Jasinska et al., 2012b), the fMRI data indicated that threat distracters had dissociable and opposite effects on the response in the cognitive-control ROIs in the difficult and easy task conditions. Namely, threat distracters acted to significantly *reduce* the response in cognitive-control regions on the more demanding incongruent MSIT trials, whereas they acted to significantly *enhance* the response in cognitive-control regions on the easier congruent MSIT trials, relative to the emotionally neutral no-distracter condition. The responses in cognitive-control regions observed in the reward-distracter condition were intermediate between threat-distracter and no-distracter conditions, but these effects did not reach significance in our data. Of note, and consistent with the behavioral results from the scanner, the difference in responses in cognitive-control regions between threat and reward distracters was also not significant.

The results of the current study contribute to a growing body of research aimed at elucidating the factors that modulate the impact of emotional stimuli on cognitive control and cognitive task performance – in other words, the factors that determine whether emotion *impairs* or *enhances* cognition. In particular, our results confirm that *task difficulty* is one factor that modulates the effects of emotional stimuli on cognitive-control processes. We found a significant interaction of task difficulty and stimulus salience, or a trend toward such interaction, in all four cognitive-control ROIs tested (left IFG/insula, ACC, DLPFC, and right IFG/insula). In all four ROIs, threat distracters reduced the response to the more demanding incongruent trials (an effect that reached significance, or a trend to significance, in left IFG/insula, ACC, and right IFG/insula), while they enhanced the response to the easier congruent trials (an effect that reached significance, or a trend to significance, in right DLPFC, left IFG/insula, and right IFG/insula).

Our finding that task difficulty modulates the impact of threat distracters on left IFG/insula response is perhaps the most intriguing. The IFG has been primarily associated with inhibitory control or response inhibition (Aron et al., 2004; Munakata et al., 2011), including inhibitory control over negative emotional stimuli (Ochsner and Gross, 2005), although it is also known to play a role in interference resolution (Nee et al., 2007). A similar pattern of modulation in left IFG was observed by Blair et al. (2007), who reported a reduction in the left IFG response to incongruent trials relative to congruent trials in the presence of threat distracters, to the point that any difference between incongruent and congruent trials was abolished. Also relevant to our study was the result obtained by Gu et al. (2012), who reported an interaction of task difficulty and stimulus salience in the left anterior insula (AI), a region anatomically adjacent to, and connected with, the left IFG. Gu et al. (2012) concluded that the AI is a key region in a network of regions that serve to integrate emotional and cognitive processes in the human brain. However, in that study, threat information increased – rather than decreased – the left AI response to the more demanding task condition (laterality judgment) relative to the easier task condition (body-part judgment). One possible explanation for this reversed direction of modulation is that *goal or task relevance* of negative emotional stimuli is another factor modulating the impact of these stimuli on cognitive-control processes (see Kanske, 2012). Indeed, previous studies suggest that task-irrelevant negative emotional distracters tend to *impair* performance on tasks engaging cognitive control (Blair et al., 2007; Jasinska et al., 2012b), whereas task-relevant negative emotional targets *enhance* performance on such tasks (Kanske and Kotz, 2011a,b). Thus, we may expect that task-irrelevant threat distracters (in the current study) and task-relevant threat targets (Gu et al., 2012) would produce an opposite pattern of modulation at the neural level as well; namely, that if threat distracters decreased the neural response in a specific cognitive-control region, threat targets should increase this neural response, and vice versa. Furthermore, both modulatory factors – task difficulty and goal relevance – may interact with stimulus salience to affect cognitive-control processes and task performance, a three-way interaction that may add further nuance and complexity to a predicted pattern of response in cognitive-control regions. To our knowledge, such three-way interaction has not yet been tested.

We also observed an interaction of task difficulty and stimulus salience in the ACC, specifically the dorsal portion of the ACC (dACC), a region well known to be involved in cognitive control (Carter et al., 1999; Botvinick et al., 2001) but not typically associated with responses to emotional stimuli. This is in contrast to the rostral ACC (rACC), which is believed to play a key role in signaling and resolving emotional conflict (Etkin et al., 2006; Egner et al., 2008), and the ventral ACC (vACC, also referred to as subgenual ACC), which has been implicated in conflict processing in the presence of emotional stimuli (Kanske and Kotz, 2011a,b). However, growing evidence suggests that the dACC, extending into the anterior midcingulate cortex (aMCC), may also be involved in integrating emotion and cognition – specifically, the integration of negative emotion, pain, and cognitive control (for review, see Shackman et al., 2011). Furthermore, the dACC, rACC, and vACC are closely related in terms of phylogeny, cytoarchitecture, and anatomical connections, with the dACC and vACC displaying a comparable high density of connections with the amygdala (Ray and Zald, 2012). Several previous studies failed to detect either main or interactive effects of stimulus salience in the dACC for negative emotional stimuli (Blair et al., 2007; Kanske and Kotz, 2011a,b; Gu et al., 2012). One possible reason is an insufficient intensity of the emotional stimuli used (e.g., a presentation of threat-related images compared to an actual pain induction; Shackman et al., 2011; Gu et al., 2012). We propose that another possible explanation for a failure to observe a main effect of stimulus salience, and particularly an interaction of stimulus salience and task difficulty, is insufficiently high level of task difficulty, which in turns produces only a modest response in the dACC, making subtle effects of modulatory factors difficult to detect.

The third cognitive-control region in which we observed an interaction between task difficulty and stimulus salience was the right DLPFC. The DLPFC is known to play a critical role in working memory (Curtis and D’Esposito, 2003), including the maintenance and updating of goal representations and task sets. A similar interaction in the DLPFC was reported by Gu et al. (2012), but that study found that threat *targets* increased the DLPFC response to the more demanding task condition relative to the easier task condition, whereas we observed that threat *distracters* reduced the DLPFC response to the harder incongruent trials compared to the easier congruent trials. Thus, as discussed above for the IFG/insula, the DLPFC response during task performance may be modulated by a three-way interaction between task difficulty, goal relevance, and stimulus salience, which is yet to be tested. Our DLPFC result also resonates with an earlier report of interactive effects – with no main effects – of induced emotional state (positive, negative, or neutral) and stimulus type (words or faces) on the DLPFC response during a working-memory task (Gray et al., 2002).

Taken together, our results suggest that negative emotional distracters can either impair or enhance cognitive control, depending on the situation, by decreasing or increasing the response in cognitive-control regions. This conclusion fits well with the view that the adaptive function of emotional states is to rapidly and flexibly switch between different modes of responding, in order to best meet the current challenges of the environment (Gray, 2004). In most situations, a goal-directed and rule-guided behavior which engages cognitive control may be most adaptive, ensuring that the organism’s needs are met; but in some situations, especially when facing a threat, it may be more adaptive to “switch off” cognitive-control processes and instead rely on fast, automatic responses to fend off danger and ensure survival. Consistent with the latter case, in addition to impairing interference resolution, cues signaling a threat of electric shock have been shown to impair response inhibition (Pessoa et al., 2012) and fearful-face distracters have been found to impair task switching (Zhou et al., 2011). From a researcher’s perspective, this generalization of threat effects across different aspects of cognitive-control presents an opportunity: because the neural mechanisms underlying the different cognitive-control processes within and across the ACC, IFG/insula, and DLPFC regions are still not well understood, emotional modulation of these regions may serve as a novel probe for elucidating these mechanisms.

We also report preliminary evidence of an interaction of task difficulty and stimulus salience on the amygdala response. Previous reports have suggested that the amygdala does not respond to cognitive conflict (Kanske and Kotz, 2011a,b) and that the amygdala response to negative emotional stimuli does not change whether these stimuli are attentional targets or distracters (Vuilleumier et al., 2001). However, evidence that the amygdala response to negative emotional stimuli is in fact modulated by attentional focus has also been reported (Pessoa et al., 2002, 2005). Furthermore, and of particular relevance to the current study, Blair et al. (2007) reported that the amygdala response can be modulated by task difficulty; specifically, in the presence of threat distracters, the left and right amygdala responses to incongruent trials were lower than to congruent trials. This is very similar to the result that we obtained in the current study: that the left and right amygdala responses to incongruent trials were lower than to congruent trials in the threat-distracter condition (although not in the reward-distracter condition). The interpretation of this pattern of results in the amygdala is at present unclear, and future studies will be needed to explain the nature and significance of the interaction between stimulus salience, task difficulty, and goal relevance on the amygdala response (for Discussion, see also Jasinska et al., 2012a).

Some limitations of the present study should be acknowledged. The first limitation is a lack of emotionally neutral distracters in our paradigm. We chose not to include such neutral distracters as a control condition, and instead to compare both threat and reward distracters against the no-distracter condition (with only neutral stimuli) and against the fixation baseline, for two reasons. Our previous behavioral study using the MSIT (Jasinska et al., 2012b), which included such closely matched neutral distracters, already established that threat distracters produced significant effects on both RTs and accuracy relative to neutral distracters as well as relative to the no-distracter condition. But the more urgent consideration was that, given a long scanning time, the amygdala response could habituate to the emotional stimuli upon repeated presentation (Breiter et al., 1996; Whalen et al., 1998b; Wright et al., 2001), which would diminish or even abolish the subtle modulation effects that we were trying to detect in the current study. Nevertheless, the lack of such neutral distracters as a control condition warrants caution in interpreting our results. In particular, it is possible that the observed effects of threat and reward distracters reflect simply distracter effects (i.e., added interference) rather than any emotion effects. Without a direct comparison between the emotional-distracter conditions and a neutral-distracter condition, we cannot be sure that the observed effects are due to the emotional salience of the distracters and not to their other attributes. However, it should be noted that if the effect was due simply to increased interference and not to emotional salience of distracters, we would expect an increase – rather than a reduction – in the response of cognitive-control regions. Another limitation of the current study is a relatively small sample size. In addition, we limited the current investigation to female participants, in order to maximize the chance of detecting the effects of interest (i.e., modulation of neural and behavioral correlates of cognitive control by threat and reward distracters) in light of considerable sex differences in emotion processing (Klein et al., 2003; Wrase et al., 2003).

An important goal for future research will be to assess the range and impact of individual differences in susceptibility to emotional distraction, conceptualized as an interplay of emotional reactivity on the one hand and cognitive-control efficiency on the other hand. The same emotional distracters may enhance cognitive control in some individuals but impair cognitive control in others, as shown with opposite patterns of behavioral performance and corresponding brain activity for working memory (Dolcos et al., 2008). Ultimately, such individual neurobiological profiles of emotion-cognition interactions may help us determine the risk of, and select the most effective treatment for, such disorders as depression, anxiety, or addiction (Dolcos et al., 2011).

In conclusion, using fMRI and a robust behavioral paradigm, we demonstrated that task difficulty modulates the impact of emotionally salient distracters on the response in cognitive-control regions, including the ACC, IFG/insula, and DLPFC, during cognitive task performance in healthy females. Specifically, threat distracters decreased the response in cognitive-control regions on the more demanding incongruent trials, whereas they increased the response in cognitive-control regions on the easier congruent trials, relative to the no-distracter condition. A similar effect was also observed in the left and right amygdala: threat distracters produced a decrease in the amygdala response on incongruent trials relative to congruent trials. These results add to our understanding of the neural processes through which emotional distracters affect cognitive control and behavior, and may have implications for the study of psychological disorders in which heightened emotional reactivity and impaired cognitive control interact to undermine normal function.

## Conflict of Interest Statement

The authors declare that the research was conducted in the absence of any commercial or financial relationships that could be construed as a potential conflict of interest.
